# Spatial Variability and Stocks of Soil Organic Carbon in the Gobi Desert of Northwestern China

**DOI:** 10.1371/journal.pone.0093584

**Published:** 2014-04-14

**Authors:** Pingping Zhang, Ming'an Shao

**Affiliations:** 1 State Key Laboratory of Soil Erosion and Dryland Farming on the Loess Plateau, Northwest A & F University, Yangling, China; 2 Key Laboratory of Ecosystem Network Observation and Modeling, Institute of Geographical Science and Natural Resources, Chinese Academy of Sciences, Beijing, China; DOE Pacific Northwest National Laboratory, United States of America

## Abstract

Soil organic carbon (SOC) plays an important role in improving soil properties and the C global cycle. Limited attention, though, has been given to assessing the spatial patterns and stocks of SOC in desert ecosystems. In this study, we quantitatively evaluated the spatial variability of SOC and its influencing factors and estimated SOC storage in a region (40 km^2^) of the Gobi desert. SOC exhibited a log-normal depth distribution with means of 1.6, 1.5, 1.4, and 1.4 g kg^−1^ for the 0–10, 10–20, 20–30, and 30–40 cm layers, respectively, and was moderately variable according to the coefficients of variation (37–42%). Variability of SOC increased as the sampling area expanded and could be well parameterized as a power function of the sampling area. Significant correlations were detected between SOC and soil physical properties, i.e. stone, sand, silt, and clay contents and soil bulk density. The relatively coarse fractions, i.e. sand, silt, and stone contents, had the largest effects on SOC variability. Experimental semivariograms of SOC were best fitted by exponential models. Nugget-to-sill ratios indicated a strong spatial dependence for SOC concentrations at all depths in the study area. The surface layer (0–10 cm) had the largest spatial dependency compared with the other layers. The mapping revealed a decreasing trend of SOC concentrations from south to north across this region of the Gobi desert, with higher levels close to an oasis and lower levels surrounded by mountains and near the desert. SOC density to depths of 20 and 40 cm for this 40 km^2^ area was estimated at 0.42 and 0.68 kg C m^−2^, respectively. This study provides an important contribution to understanding the role of the Gobi desert in the global carbon cycle.

## Introduction

Soil organic carbon (SOC) has an important influence on the physical, chemical, and biological properties of soil and is critical for improving soil fertility and quality, increasing the water holding capacity of soil, reducing soil erosion, and enhancing crop productivity [1], [2]. With climate change and environmental issues dominating global concerns, SOC has received increasing attention worldwide because of its important role in the global C cycle and its potential feedback on the global warming [3]–[6]. As one of the largest and most dynamic component in the global C cycle, the SOC stock is at least two times the amount of C stored in the vegetation and atmosphere [7]. Thus, a small loss of SOC pool due to changes in fertilization, cropping system, farming practices, and soil erosion could significantly increase the atmospheric CO_2_ [8]–[11]. On the other hand, soils can increase the existing SOC pool by sequestration of C from the atmosphere [12]–[15], the processes of which are an active area of study. Reliable assessment of the spatial patterns and stocks of SOC at one timeline as a baseline is essential for understanding the potential of soils to sequester C, for quantifying the SOC sink or source capacity of soils in changing environments, and for developing the strategies necessary to mitigate the effects of global warming [16], [17].

In recent years, extensive work has been conducted toward estimating the SOC stocks and distribution patterns at the global, continental, country, and regional scales [11], [18]–[24]. For example, the global SOC stock has been estimated to be about 2400 Pg C in the top 2 m [4]. However, these estimations are highly uncertain because of the gaps in spatial coverage for many regions that causes difficulties to develop a harmonized SOC baseline [22]–[24]. In addition, the selection of the type of SOC database, the land use and/or soil map, the mapping resolution, reference depth, bulk density or other information can also have a great effect on the final SOC stock estimation [25]. Similarly, due to inconsistent estimation methods and limited data, the SOC stock estimations in China are also uncertain and has varied greatly, from 50 to180 Pg, and SOC density from 54.6 to 190.5 t C/ha [22]. The accuracy of these large-scale SOC stock estimations largely depends on the data availability from site-based or small-scale measurements [6], [24]. To reduce the uncertainty of SOC stocks estimation and better understand the role of SOC in the global C cycle, reliable baseline datasets providing information on SOC stocks in all types of sites and ecosystems are necessary.

Desertification is one of the most severe types of land degradation in arid and semiarid areas of the world [26]. Due to the harsh natural conditions and the fragile ecological environment, desert ecosystems are more sensitive to climate change, leading to the emission of CO_2_ to the atmosphere and a reduction in the pool of SOC [27], [28]. In contrast, it is possible to increase SOC concentrations in desert soils through the adoption of restorative measures such as the establishment of plants [14], [29], [30] and the prohibition of grazing [31]. [32] indicated that the control of desertification could globally sequester 0.9–1.9 Pg C yr^−1^ over a period of 25–50 years.

China is also seriously threatened by desertification [33], [34]. [27] estimated that desertified land in China potentially covers 158 Mha, comprising 81 Mha of slight, 61 Mha of moderate, and 35 Mha of severe desertification. The widely distributed desertified lands in China thus likely have a considerable effect on the regional terrestrial C balance and the feedbacks that affect climate change [35]. Although some studies have been conducted on assessment of SOC concentrations/stocks in desert area, many questions still remain open. So far, most studies on SOC stock estimates from desert ecosystems have been carried out in sandy desert [36]–[39], and only few from the Gobi desert are available [40]. Due to the difficulties and associated costs of soil sampling, most studies estimating SOC stocks rely on information taken from a relatively small number of representative sampling points or profiles [41]. This decreases the estimation accuracy of SOC stocks in desert ecosystems and limits our capability to evaluate the C budget, to assess its contribution to the increasing global concentrations of CO_2_, and to propose measures to increase the sequestration of organic C in soils. Therefore, to better understand the SOC pool in desert environments, it is necessary to conduct more intensive site and local scale estimates of the variance in SOC, and the possible spatial controls on this variance structure.

Gansu province is one of the main desertified areas induced by wind and one of the source regions of sandstorms in northern China. In this paper, a typical fenced region of the Gobi desert was chosen as a study case. The objectives of this study were: (1) to estimate SOC concentrations and determine the spatial distribution of SOC, (2) to analyse the relationships between SOC concentrations and environmental factors, and (3) to estimate SOC density and storage in the study area.

## Materials and Methods

### Ethics statement

The study area belong to Linze Inland River Basin Research Station (39°21′ N and 100°07′E,1389 m), a department of the Cold and Arid Regions Environmental and Engineering Research Institute, Chinese Academy of Sciences. The study was approved by the Cold and Arid Regions Environmental and Engineering Research Institute, Chinese Academy of Sciences.

### Study area

**Figure 1:**
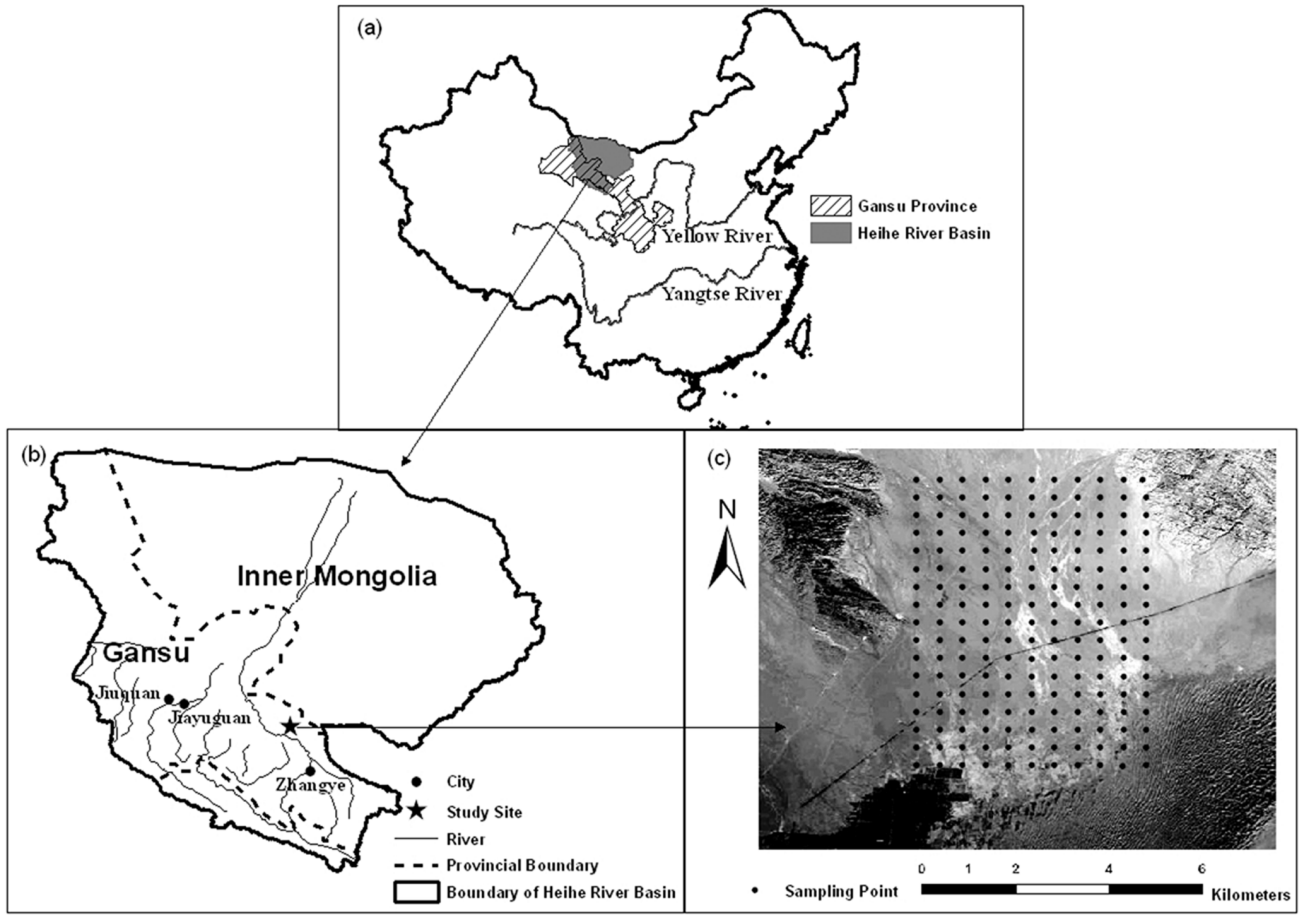
The location of Gansu Province and the Heihe River Basin, China (a), the study site in the Heihe River Basin (b), and the soil-sampling points in the study area (c).

The study area, occupying approximately 40 km^2^ (5 km×8 km), is located in the Gobi desert in the middle of Gansu province (the central reaches of the Heihe River Basin) of Northwestern China, between latitudes 39°24′ and 39°28′N and longitudes 100°08′ and 100°11′E (Fig. 1*a,b*). The region is a relatively flat alluvial plain (elevation ranging from 1390 to 1470 m) bordered by a young oasis to the southwest, the remnants of the Qilian Mountains to the north, and an extension of the Badain Jaran Desert to the southeast (Fig. 1*c*). The area is characterized by low and seasonal variability in rainfall and is classified as a typical temperate desert. The mean annual precipitation and air temperature are 117 mm and 7.6°C, respectively. Rainfall in brief summer showers contributes 65% of the annual total precipitation. The mean annual pan-evaporation is approximately 2390 mm, twenty times greater than the annual precipitation. The average annual wind speed is 3.2 m s^−1^, with the resultant wind coming from the northwest, and the dominant windy days and wind storms occur between March and May [42]. The zonal soil is classified as gray-brown desert soil, derived from gravelly diluvial-alluvial materials of the denuded monadnock [43]. Stones are present in a significant proportion of the surface and sub-soil horizons. The aboveground plant cover is discontinuous and can be described as patches of sub-shrubs surrounded by bare areas. The study area has been fenced and is protected from grazing for the purpose of revegetation and reclamation. The dominant plant species are *Nitraria sphaerocarpa* Maxim. and *Reaumuria soongorica* (Pall.) Maxim., and the accompanying plant species are mainly *Kalidium gracile* Fenzl., *Allium mongolicum* Rgl., *Bassia dasyphylla* (Fisch. and Mey.) Kuntze. and *Halogeton arachnoideus* Moq.

### Soil sampling and laboratory analysis

Soil samples were collected from a total of 187 locations on a regular grid of 500 m×500 m throughout the study area from August to September in 2011. A portable GPS receiver (Garmin GPSmap 62 s) was used to locate the sampling site, as displayed in Figure 1*c*. At each location, soil was collected from four depths (0–10, 10–20, 20–30, and 30–40 cm) at five randomly selected sampling points within a radius of approximately 20 cm. The five sub-samples were then combined to produce one representative sample. In total, 748 soil samples were collected. The samples were all air dried, weighed, and sieved to 2 mm to separate the coarse (>2 mm) and fine (<2 mm) fractions. The former was reweighed to determine the stone content. The latter was separated into two parts: one was subject to further particle-size analyses by laser diffraction using a Mastersizer 2000 (Malvern Instruments, Malvern, England), and the other was ground to pass through a 0.25-mm mesh for SOC concentration analysis. The SOC concentration (g kg^−1^) was measured using the potassium dichromate-wet combustion method [44]. Undisturbed soil samples cores (100 cm^3^) were also collected for determining soil bulk density (BD) in each layer. To reduce the influences of stones on BD measurement, we averaged five replicate measurements for each location and layer.

### Re-sampling method

Estimating SOC variability at different scales is important for effective soil survey sampling design and SOC change prediction [2]. In this study, to detect the change tendency of SOC variability with the size of the sampling area, a series of sampling point allocations with different areas were done through re-sampling using all sampling points (n = 187) in the study area (5 km×8 km) [45], [46]. For convenience, the west-east and south-north distances of the re-sampling area were set integral multiples of 1 km to generate five re-sampling options for the west-east distance and eight options for the south-north distance. The random combination of these two sets of options thus produced 40 potential re-sampling methods. Some of these methods, though, were the same within the study area (e.g., the re-sampling methods of 2 km×6 km, 3 km×4 km, and 4 km×3 km yielded the same area), so finally a total of 24 kinds of re-sampling areas were obtained (Table 1). SOC variability at a certain area was computed by averaging the CV values of all the possible re-sampling scenarios with the same area.

**Table 1:** Details of the re-sampling areas and sampling methods.

Re-sampling area (km^2^)	Sampling method	Re-sampling area (km^2^)	Sampling method
1	1×1*	15	3×5; 5×3
2	1×2; 2×1	16	2×8; 4×4
3	1×3; 3×1	18	3×6
4	1×4; 4×1; 2×2	20	4×5; 5×4
5	1×5; 5×1	21	3×7
6	1×6; 2×3; 3×2	24	3×8; 4×6
7	1×7	25	5×5
8	1×8; 2×4; 4×2	28	4×7
9	3×8	30	5×6
10	2×5; 5×2	32	4×8
12	2×6; 3×4; 4×3	35	5×7
14	2×7	40	5×8

*The digit before the multiplication sign represents the west-east sampling distance (km), and the digit after it represents the south-north sampling distance (km).

### Calculation of SOC density and stock

The SOC density of a single layer was estimated based on Eq. (1):
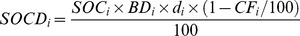
(1)where SOCD_i_ and SOC_i_ are SOC density (kg C m^−2^) and concentration (g kg^−1^) of the i^th^ layer, BD_i_ is the bulk density of the i^th^ layer (g cm^−3^), d_i_ is the depth of the i^th^ layer (cm), and CF_i_ is the fraction (%) of coarse fragments >2 mm in the i^th^ layer.

For an individual profile with a depth D (cm), SOC densities were then calculated by summarising the SOC density of each soil layer i:
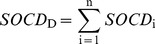
(2)The total SOC storage is calculated as:
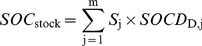
(3)where S_j_ is the total area (m^2^) of a given land-use type j and SOCD_D,j_ is the average SOC density of the j^th^ land-use type (kg m^-2^).

### Statistical analysis

A descriptive statistical analysis was first used to illustrate the central trend and the overall variation of the variables. This analysis included descriptions of the minimum, maximum, mean, median, skewness, Kurtosis, standard deviation (SD), and coefficients of variation (CVs). A one-sample Kolmogorov-Smirnov (K-S) test was used to examine the normality of the data, and natural logarithmic transformations were performed where necessary to meet the normality requirement of geostatistical analysis. The means of different layers were compared by a one-way analysis of variance (ANOVA). Correlation analysis and stepwise linear regression analysis were performed to understand relationships between SOC concentrations and the environmental factors. All statistical analyses used the programme SPSS v. 16.0 (SPSS Inc., Chicago, IL, USA).

Geostatistical methods such as semivariogram calculation, cross-validation, kriging, and mapping have been widely applied in the study of SOC spatial distribution [47], [48]. Semivariograms were used to determine the degree of spatial dependence. Before semivariogram calculation, a preliminary semivariogram surface analysis was performed to detect any zonal effect or trend in direction [49]. The experimental semivariogram, 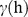, is half the expected squared difference between paired data separated by a distance h and is expressed as:
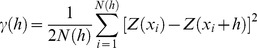
(4)where 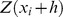 and 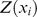 are observations at positions 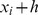 and 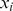, respectively, and 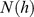 denotes the number of data pairs separated by the lag distance, h. A semivariogram model contains three important parameters which interpret the spatial structure of soil properties: nugget (C_0_), sill (C+C_0_), and range (A). Nugget represents the undetectable measurement error, inherent variability or the variation within the minimum sampling distance. Sill is the upper limit of the semivariogram model, representing the total variation. The separation distance at which the sill is reached is the range of spatial dependence. Samples separated by distances smaller than the range are spatially related, whereas samples separated by larger distances are not spatially related. The nugget ratio (C_0_/C_0_+C) can be regarded as a criterion for classifying the spatial dependence of soil properties. A variable is considered to have strong, moderate, or weak spatial dependence if the ratio is less than 0.25; between 0.25 and 0.75; and over 0.75, respectively [50].

In this study, all semivariograms were estimated with fixed distance intervals of 500 m, and the maximum distance was set to 4700 m. Because 4700 m is less than half the maximum distance between sampling sites, it coincides with the requirement of geostatistical analysis [51]. A total of nine sets of class intervals were generated. There are several commonly used semivariogram models (such as spherical, exponential, Gaussian, and linear models). The best model was based on two criteria: small residual sum of squares (RSS) and high coefficient of determination (R^2^). The best-fit parameters were subsequently estimated using weighted least squares regression method.

After selecting the best-fit semivariogram models, ordinary kriging was used as an interpolation method to predict values for SOC concentrations. The prediction was calculated as the linear sum:
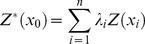
(5)where 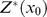
is the value to be predicted at the location 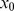, 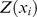 is the known value at the sampling location 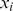, n is the number of locations within the search neighbourhood used for the prediction, and 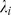 is the kriging weight assigned to 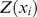.

The predicted map quality of the SOC was tested by cross-validation with replacement. Three indices were calculated to assess the effectiveness of kriging: mean error (ME), root mean square error (RMSE), and root mean square standardised error (RMSSE) [52].

The geostatistical analysis was performed with GS+ v. 7.0 (Gamma Design Software, Plainwell, Michigan, USA), and contour maps through ordinary kriging were produced with GIS software ArcView v. 3.3 and its extension module of Spatial Analysis v. 2 (ESRI Inc., Redlands, California, USA).

## Results and Discussion

### Soil physical properties

Due to the lack of mineral weathering and to long-term wind and water erosion, this natural desert exhibited a high content of stones, with an average of 27.42% stones in the top 40 cm of soil. The stone content increased slowly from 12.2% in the 0–10 cm layer to 15.2% in the 20–30 cm layer, and then abruptly increased to 69.1% in the 30–40 cm layer (Table 2). All soil layers, except for 30–40 cm, contained more than 70% sand and much less silt and clay. Surface layer (0–10 cm) tended to have a higher content of clay (*P*<0.01), perhaps due to erosion. Clay contents tend to increase at shallower depths as the amount of soil erosion increases [53]. Beyond that, other soil properties (sand, silt, and BD) remained constant throughout the entire soil profile. Compared with the lower variability of sand (CVs of 19.6–22.1%), stone (CVs of 24.5–100.5%), silt (CVs of 85.5–111.9%) and clay (CVs of 84.4–123.0%) showed moderate or strong variability. The CV of the BD at each depth was ≤10.5%, indicating that BD was not very variable throughout the study area. The CVs indicated that the variabilities of all constituent contents, except for stone content, generally increased with soil depth.

**Table 2:** Selected soil physical properties at different soil depths.

Variables	Statistical parameter	Soil depth			
		0–10 cm	10–20 cm	20–30 cm	30–40 cm
Stones	Mean (%)	12.1 a	13.3 ab	15.2 b	69.1 c
	CV (%)	100.5	103.4	101.3	24.5
Sand	Mean (%)	70.9 a	72.4 a	71.1 a	26.0 a
	CV (%)	20.1	19.7	21	22.1
Silt	Mean (%)	7.5 a	6.5 a	6.5 a	2.4 a
	CV (%)	85.5	97.5	107.5	111.9
Clay	Mean (%)	9.5 a	7.8 ab	7.2 b	2.6 b
	CV (%)	84.4	102.4	111.3	123
BD	Mean (g cm^−3^)	1.4 a	1.4 a	1.4 a	0.5 a
	CV (%)	8.8	10.4	9.7	10.1

BD, bulk density; CV, coefficient of variation; Stones, >2 mm; Sand, 2–0.2 mm; Silt, 0.2–0.002 mm; Clay, <0.002 mm. Mean values followed by the same letter are not significantly different (α = 0.05) at different soil depths using the LSD method.

### SOC concentration and its variability

The calculation of variation function generally should be in accord with normal distribution, otherwise it may cause the proportional effect, raising the sill or nugget values [54]. As shown in Table 3, the raw SOC exhibited a positively skewed distribution in this study area. Thus, we used the logarithmic transformation to reduce the data skewness. The Ln-transformed data for all four layers passed the Kolmogorov-Smirnov test at a significance level higher than 0.05 (not shown) and consequently could be used in the analysis of the geostatistical variation function.

**Table 3:** Summary of statistical parameters for soil organic carbon (SOC) concentrations at different soil depths.

Variables	SOC (g kg^−1^)			
	0–10 cm	10–20 cm	20–30 cm	30–40 cm
Maximum	4.5	3.9	4.3	3.6
Minimum	0.4	0.5	0.5	0.5
Mean	1.6 a	1.5 ab	1.4 b	1.4 b
SD	0.6	0.6	0.6	0.6
CV	36.8	38.5	41.0	42.1
Skewness	1.83	1.26	1.64	1.15
Kurtosis	5.95	2.40	4.66	1.04
*P* of K-S test	0.001	0.012	0.009	0.006

SD, standard deviation; CV, coefficient of variation. Mean values followed by the same letter are not significantly different (α = 0.05) at different soil depths using the LSD method.

SOC concentrations were generally variable, ranging between 0.4–4.5 g kg^−1^, 0.5–3.9 g kg^−1^, 0.5–4.3 g kg^−1^, and 0.5–3.6 g kg^−1^ for the four descending layers, respectively (Table 3). According to the soil-nutrient classification standards from the Second National Soil Survey in China [55], the SOC level in this area was very low; only seven of the 748 soil samples were above the lowest classification standard (3.5 g kg^−1^). The mean were 1.6, 1.5, 1.4, and 1.4 g kg^−1^ for the 0–10, 10–20, 20–30, and 30–40 cm layers, respectively, and progressively decreased with soil depth. The surface layer (0–10 cm) had the highest SOC concentrations, due to inputs of organic material that accumulated from organic litter and root residues [25], [56], and higher soil aeration enabled higher soil enzyme activities in the surface layer than in the deeper layers [29]. Some researchers have also reported that SOC content decreased with depth in other natural ecosystems [6], [10], but this trend is unlikely at sites with substantial human intervention such as orchards and tree nurseries [6]. In these sites, the topsoil may not have SOC contents much higher than the underlying layers, as a result of the management practices, such as heavy tillage, that could have enhanced SOC runoff and soil respiration [6].

The CVs of SOC concentrations varied from 36.8% to 42.1%, which are considered as moderate variation [57]. A moderate variability of SOC has also been reported in other studies at multiple scales [11], [25], [41], [58]. [11], [25], [58] reported a decreasing trend of SOC variability with increasing soil depth. Our study, however, found the opposite trend. This can probably be explained by the different land-use types and vegetation characteristics. In their studies, croplands, grasslands, and forestland are the main land-use types. SOC is thus greatly affected by human activities, such as grazing and deforestation, and the agricultural managements of plowing, fertilisation, harvesting, and crop rotation which can have a greater impact on the surface soil layer than on the deeper layers [2], [6], [31], [59]. Moreover, the high vegetation coverage in these land-use types indicates a high biomass of plant roots and litter, and an active soil microbial and enzyme activities, which mainly occur in the surface soil layer [29], [56]. By contrast, the natural desert region in our study is rarely disturbed by human activities and the vegetation growth is limited by inferior soil and water conditions. The surface layer is more susceptible to processes, such as sedimentation and erosion, which can homogenise the distribution of SOC [60]. Therefore, SOC in the deeper layer tend to have greater spatial variability. Additionally, the preferential transport of SOC via cracks during dry periods could further increase the heterogeneity of SOC in the deeper soil horizons [10].

### Response of SOC variability to the expansion of area

**Figure 2:**
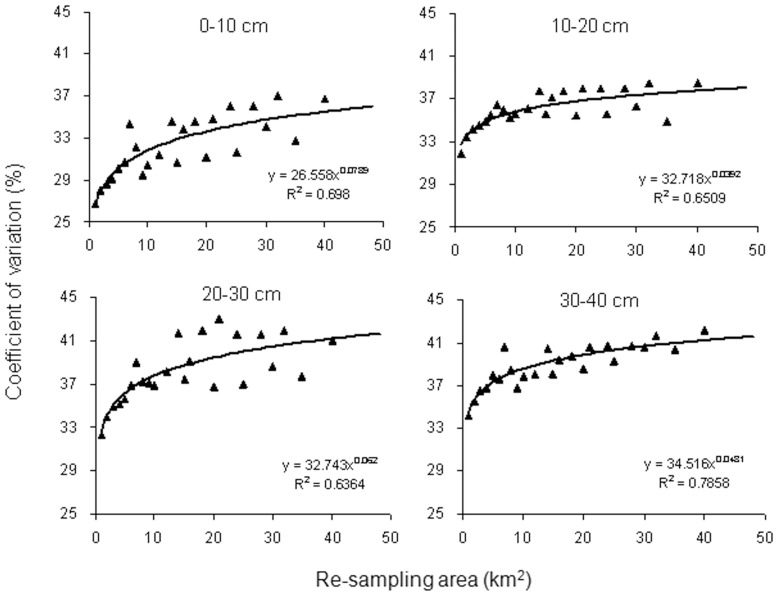
Coefficient of variation of soil organic carbon (SOC) concentrations at different re-sampling areas.

Fig. 2 demonstrated that CV of SOC concentration increased with the expansion of area and could be well parameterized as a power function of the sampling area for all the four soil layers. The factors influencing SOC concentration variability are scale dependent. For example, parent material, precipitation and geological history are of major importance to affect SOC at large scales. However, microtopography (such as the run-off gullies) and vegetation may be the dominant factors of SOC variability at small scales [61]. As sampling area increases, the origin of SOC variations above may get increasingly complex and heterogeneous, contributing to greater variability [2]. Moreover, the value of the fractal power parameter in the surface layer (0.0789) was much larger than that in the other three layers (0.0392–0.0620), indicating SOC concentration variability in the surface layer was more sensitive to the expansion of area. If increasing the same area, the SOC variability will increase more in the surface layer than that in the deeper layers.

The function between CV of SOC concentration and sampling area can also be used to estimate the variability of SOC concentration at a desired area and, consequently, the number of required samples (NRS). In order to obtain the mean value of SOC concentration with an accuracy level of Δ, at a confidence level of 1-α, the sample size should reach the requirement of NSR = 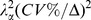
(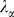 is the value of the Student's t-distribution at the confidence level of 1-α) [48]. Take the surface layer (0–10 cm) for example, NRS is found to be equal to 110, 227 and 326 for an area of 1, 100 and 1000 km^2^, respectively, by assuming an accuracy level of 5% and a confidence level of 95%.

### Pearson correlation and stepwise linear regression analyses

Under the extremely arid climate, the vegetational cover in this region of the Gobi desert is very low, and the chemical and biotic influences on soil development are relatively minor. Climate, soil type, and terrain can be disregarded as variables considering the small size and flatness of the study area. The physical properties of the soil were thus most likely responsible for the SOC variability in our study area.

Table 4 shows the correlation analyses between the soil physical properties and SOC concentrations. The four soil layers had similar patterns. SOC concentrations were positively correlated with both silt and clay contents and were negatively correlated with stone and sand contents and BD. The positive effects of clay and silt contents on SOC concentrations are likely due to the ability of clay and silt particles to adsorb organic matter. Finer particles are better than larger particles in protecting bound organic matter for longer times [62]. Moreover, the distribution of soil particle size and BD can indirectly impact SOC dynamics by affecting the physical structure, drainage, and aeration of soils [63].

**Table 4:** Correlation analysis between soil organic carbon (SOC) concentrations and soil physical properties at different soil depths.

Variables	SOC (g kg^−1^)			
	0–10 cm	10–20 cm	20–30 cm	30–40 cm
Stones (%)	−0.196**	−0.201**	−0.201**	−0.448**
Sand (%)	−0.679**	−0.605**	−0.572**	−0.564**
Silt (%)	0.676**	0.606**	0.572**	0.567**
Clay (%)	0.663**	0.593**	0.561**	0.552**
BD (g cm^−3^)	−0.413**	−0.387**	−0.432**	−0.398**

BD, bulk density; ** denotes significance of correlation at *P*<0.01.

The stepwise linear regression analysis was further performed to delineate the effect of different factors on SOC and to find the best predictive variables for SOC. A summary of these linear models is shown in Table 5. For the surface layer (0–10 cm), the regression model explained 47.0% of the overall SOC variability in which most of the variability was attributable to sand (39.7%) and stone contents (7.3%). For the other three layers, however, silt and stone contents together accounted for approximately 35% of the total variance of SOC concentrations. These results indicated that the relatively coarse fractions (stones, sand, and silt) were more important for the explanation of variability in SOC concentrations in this study area

**Table 5:** Stepwise multiple linear regression of soil organic carbon (SOC) concentrations with selected soil variables at different soil depths.

Soil depth	Independent variables	Coefficient	Explained Variance (%)
0–10 cm	Constant	3.589 **	
	Sand	−2.617 **	39.78
	Stones	0.006 **	7.30
	Adjusted *R^2^*	0.470	
	MSE	0.417	
10–20 cm	Constant	0.989 **	
	Silt	5.200 **	31.18
	Stones	0.006 **	6.52
	Adjusted *R^2^*	0.377	
	MSE	0.444	
20–30 cm	Constant	0.950 **	
	Silt	4.599 **	27.20
	Stones	0.006 **	6.80
	Adjusted *R^2^*	0.340	
	MSE	0.466	
30–40 cm	Constant	1.613 **	
	Silt	3.206 **	24.20
	Stones	−0.007 **	10.00
	Adjusted *R^2^*	0.342	
	MSE	0.477	

MSE, mean squared error; *R^2^*, coefficient of determination; ** Denotes significance of correlation at *P*<0.01.

### Semivariogram and parameters

Spatial structure was not significantly associated with direction in the study area. Only isotropic semivariograms were thus plotted for SOC concentrations by using the model best fitted by the least squares regression method. Exponential models were theoretically optimal for all four soil layers.

**Figure 3:**
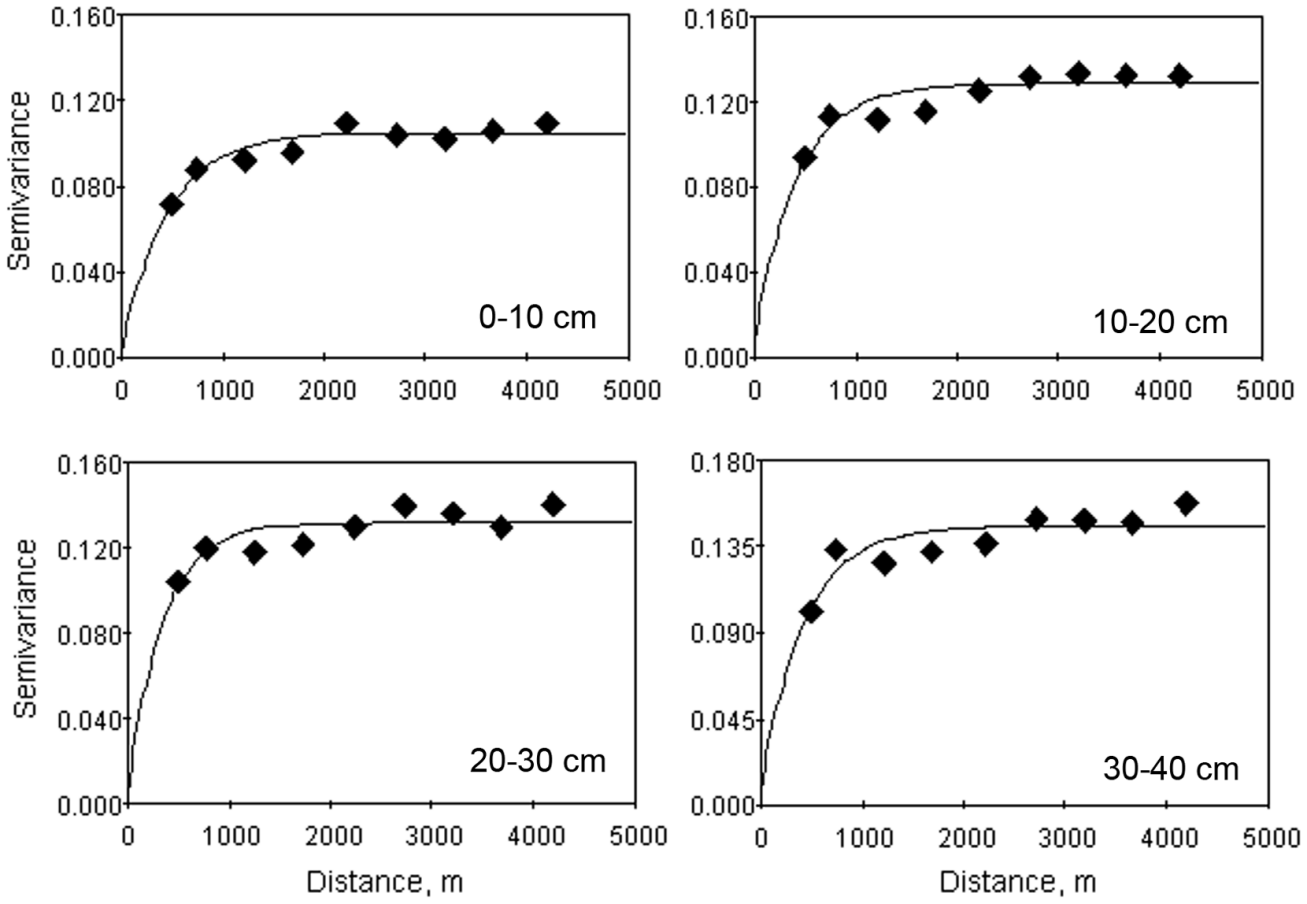
Isotropic semivariograms of soil organic carbon (SOC) concentrations at different soil depths.

The semivariogram models and best-fitted model parameters of SOC concentration at different soil depths are given in Fig. 3 and Table 6. The semivariogram of the SOC concentrations indicated a slightly smaller nugget effect (C_0_) in the surface soil layer (0–10 cm) than in the other three layers, implying that the deeper soil layers had higher undetectable experimental error, short-range variability, and random and inherent variability of SOC concentration than the surface soil layer [3]. The sill values, representing total variation, showed an increasing trend from the surface soil layer to the deepest layer, which further validated the results obtained by conventional statistical methods. The nugget ratio (C_0_/C_0_+C) ranged from 0.018 to 0.054, indicating a strong spatial dependence for SOC concentrations for all four soil layers in our study [50]. Strong spatial dependency of soil properties can usually be attributed to intrinsic factors. In this natural desert, fenced enclosures for natural restoration have been implemented for many years and are rarely disturbed by human activities and grazing, so we suggest that the variability of SOC concentrations in this region may be highly dependent on the mineralogical composition of the parental material and on the weathering processes that have led to its formation [64]–[66].

**Table 6:** Parameters of the semivariogram models estimated for soil organic carbon (SOC) concentrations at different soil depths.

Soil depth	Model	Nugget C_0_	Still (C_0_+C)	C_0_/(C_0_+C)	Range (m)	*R* ^2^
0–10 cm	exponential	0.0019	0.1048	0.018	1347	0.884
10–20 cm	exponential	0.0069	0.1288	0.054	1251	0.770
20–30 cm	exponential	0.0047	0.1324	0.035	1047	0.681
30–40 cm	exponential	0.0069	0.1458	0.047	1254	0.724

*R^2^*, coefficient of determination.

The range also changed significantly with soil depth, which is likely due to the control of the distribution pattern of SOC by different soil processes. The range for the 0–10 cm layer was the largest, indicating a larger spatial autocorrelation in the surface soil layer than in the deeper soil layers. The surface soil layer is most sensitive to erosion, which could homogenise the distribution of SOC and increase the spatial autocorrelation distance [60]. [67] also showed that wind erosion significantly changed the spatial distribution patterns of SOC over two or three windy seasons. With the increasing of soil depth, the influence of erosion on SOC distribution was weakened, and the SOC tended to be much more independent and was characterized by a stochastic pattern. As shown in Table 6, the range decreased from 1347 to 1047 m from 0–10 cm to 20–30 cm. The range in the 30–40 cm layer, however, increased slightly, reflecting the influence of parental materials on the spatial structure of SOC in deeper layers. In deeper soil layers, the SOC was probably inherited from the soil parent materials because the soils are young with little weathering or anthropogenic impacts [65]. Since the parental materials are distributed quite uniformly across the study area [66], it leads to a better distribution of spatial continuity. Because the range was larger than our sampling interval (500 m), our sampling system was sufficiently robust to detect spatial relationships on the scale of the landscape.

### Kriging of spatial variation of SOC concentrations

**Figure 4:**
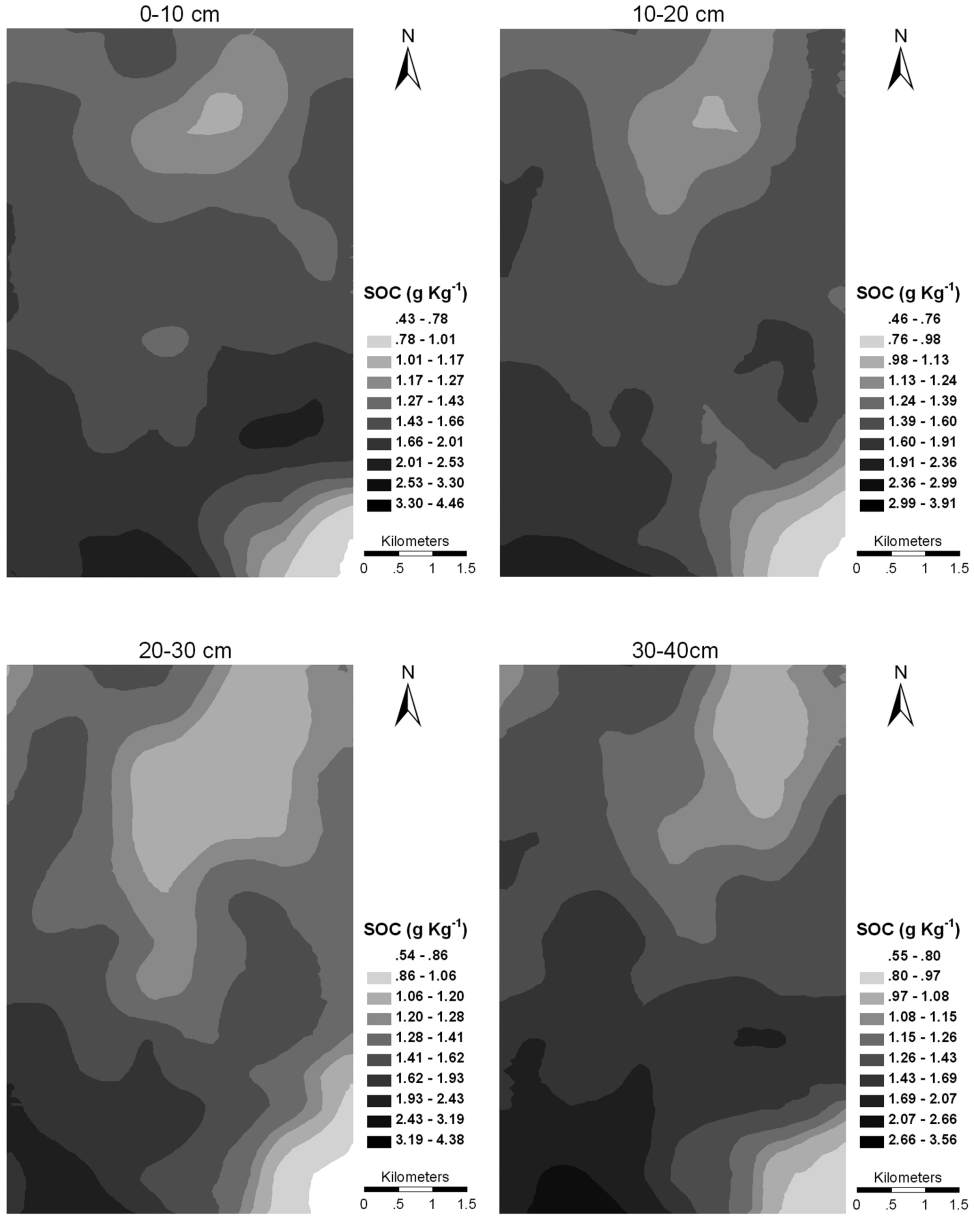
Distribution of soil organic carbon (SOC) concentrations across the study area.

The semivariogram models were used as input to ordinary kriging, and the resulting distribution maps are shown in Fig. 4. The interpolation cross-validation was carried out to test the effectiveness of the prediction maps, and the associated prediction errors for each map are shown in Table 7. ME determined the degree of bias in the estimates and should be close to zero. RMSE quantified the average differences between prediction and observation and should be as small as possible. If the model accurately described the data, RMSSE should be close to unity. Based on the above criteria, the predicted maps of SOC concentration for the study area were reliable.

**Table 7:** Verification of interpolation reliability for soil organic carbon (SOC) concentrations at different soil depths.

Soil depth	ME	RMSE	RMSSE
0–10 cm	−0.00821	0.535	1.105
10–20 cm	−0.00083	0.544	1.056
20–30 cm	0.00173	0.504	1.017
30–40 cm	−0.00143	0.570	1.104

ME, mean error; RMSE, root mean square error; RMSSE, root mean square standardized error.

The character of SOC distribution is clearly similar for each layer, and the entire study area is characterised by low concentrations of SOC and high variation. SOC concentrations generally decreased gradually from south to north. The southwest part of the study area, close to the oasis, tended to have higher concentrations of SOC. The shelter belts in front of the oasis functioned as natural barriers to reduce wind velocity and intercept the fine material, which tends to be rich in SOC [68]. The increased fine fractions also improved the soil properties, encouraging the colonisation of some annuals, e.g. *B. dasyphyll*a and *H. arachnoideus*. Their rapid growth and death provided an important influx of SOC. In contrast, relatively low concentrations of SOC were mainly distributed in the north and the southwest. The northern part of the site was surrounded by mountains and was characterised by coarse soils and little vegetations, facilitating the drifting of fine particles and a noticeable decline in SOC. On the other hand, the southwestern region was linked with the Badain Juran desert and the longtime encroachment of drifted sand from the desert produced a more sandy texture in this area.

### SOC density and stocks

SOC density is indispensable for the assessment of SOC stocks and is the required measurement of account for the Clean Development Mechanism of the United Nations Framework Convention on Climate Change [69]. SOC density was 0.22, 0.20, 0.19, and 0.07 kg C m^−2^ for the 0–10, 10–20, 20–30, and 30–40 cm layers, respectively, and dropped sharply with increasing depth due to the high stone content. The low value in the 30–40 cm layer indicates that the SOC density would be even lower below 40 cm because of the increasing number of stones. We can thus reasonably conclude that SOC in this region of the Gobi desert was mainly stored in the upper 30 cm of soil. The overall SOC stocks in the upper 20 cm was 0.42 kg C m^−2^ and to a depth of 40 cm 0.68 kg C m^−2^. When compared to the reported values for other regions in China [11], [25], [70], [71], [72], due to natural drought, large number of stones, and intense soil erosion, SOC stocks in the Gobi desert region was very low. However, since the Gobi desert (568,980 km^2^) accounts for about 5.9% of China's total territory [73] and is sensitive to climate change [17], it is likely to have a considerable effect on the terrestrial C balance in China.

Table 8 summarises the results of SOC studies (including SOC concentrations and densities) in other desert regions in China. The SOC concentrations and densities in our study area were generally in the same range as those in other regions, except that SOC concentrations were a little higher than those in the Erdos and Aershan regions. Different reference soil depths, different sampling methods, and the higher patchiness of our study area may account for our higher SOC concentrations. Even though the soils in our study area had higher fractions of stones, patches with more fertile soil still allowed the growth of vegetation, which thus increased the SOC concentrations. Compared with some of the other regions mentioned in Table 8, SOC in our study site is less affected by grazing, which can give rise to a considerable decrease in ground coverage and primary productivity, and thus accelerate soil erosion by wind and result in loss of SOC [74]. However, the improvement of soil quality through the adoption of grazing prohibition can increase SOC concentrations [36]. Moreover, higher SOC concentrations in our study site can also be attributed to the reduced mineralization rate of SOC, because of the lack of water in this region [66]. Comparisons among the studies listed in Table 8, however, remain limited due to differences in sampling methods of SOC measurement and reference soil depths. Identifying the dynamics of SOC in changing desert environments is difficult. We should thus develop more site inventories of SOC in desert environments or use a comparable approach to better understand the potential changes of SOC in desert environments, which will lay the groundwork for developing more effective strategies to combat soil desertification and reduce the risk of desertification in the future.

**Table 8:** Comparison of SOC concentrations and SOC stocks in other desert areas in China.

Region	Environment	Mean annual precipitation (mm)	Area (km^2^)	Reference depth (cm)	SOC concentration (g kg^−1^)	SOC stock (kg C m^−2^)	Reference
Erdos, Inner Mongolia	Temperate shrub desert	170 (161–209)	Profile measurement	0–300	0.42		[75]
Aershan, Inner Mongolia	Temperate desert	102 (61–121)	Profile measurement	0–300	0.25		[75]
Alxa league, Inner Mongolia	Desert steppe	134	Point measurement (sites with different grazing degrees)	0–40	1.73–2.05		[74]
Horqin Sand Land, Inner Mongolia	Desert grassland	350–450	Point measurement (sites with different desertification types and degrees)	0–30	0.37–4.35	0.19–1.76	[38]
Alxa Left County, Inner Mongolia	Desert shrubland	60–160	2.5	0–30		0.21–2.80	[39]
Horqin region, Inner Mongolia	Sand Land (sand dunes)	360	Point measurement (grazed and restored sites)	0–20	1.31–2.03		[37]
Horqin region, Inner Mongolia	Sand Land (mobile dunes)	360	Point measurement (sites with different restoration processes of dune vegetation)	0–20	0.68–1.29		[77]
Hunshandake, eastern Inner Mongolia	Sandy Land	300 (165–572)	21 400	variable	0.44–4.37		[76]
Central Gansu province	Gobi desert	117	40	0–40	1.45 (0.43–4.46)	0.68	This study

## Conclusions

We have provided estimates of spatial SOC concentrations and stocks for this region of the Gobi desert that are more accurate than previous estimates. Classical statistics indicated that SOC concentrations decreased with increasing soil depth and were moderately variable in the study area. The deepest soil layer (30–40 cm) had the highest amount of variation in SOC concentrations. Significant correlations were detected between SOC and selected physical properties of the soil, especially the stone, sand, and silt contents. The composition of the parental material (such as the distribution of soil particle size) and the weathering (such as erosion and sedimentation) that led to its formation may be responsible for the strong spatial dependence of SOC. This dependence implies that SOC in desert ecosystem is sensitive to climate change and thus represents an important dynamic pool of C in the global C cycle. The kriging interpolated maps indicated a decreasing trend of SOC concentrations from south to north across the study area, which was apparently related to the location of the study area. This study contributes to our understanding of the role of Gobi desert ecosystem in the global C cycle and incorporation of small-scale spatial variations of SOC into large-scale spatiotemporal models.
